# Structural insights into Cas9 inhibition by AcrIIA17 via bridge helix interaction

**DOI:** 10.1016/j.isci.2026.117218

**Published:** 2026-08-19

**Authors:** Gi Eob Kim, Hyo Been Jin, Yong Jun Kang, Hyun Ho Park

**Affiliations:** 1 College of Pharmacy, Chung-Ang University, Seoul 06974, Republic of Korea; 2 Department of Global Innovative Drugs, Graduate School of Chung-Ang University, Seoul 06974, Republic of Korea

**Keywords:** anti-CRISPR, AcrIIA17, adaptive immunity, CRISPR-Cas system, crystal structure

## Abstract

Anti-CRISPR (Acr) proteins have evolved in bacteriophages and mobile genetic elements to counteract CRISPR-Cas immune systems through diverse inhibitory mechanisms. Here, we present the crystal structure of AcrIIA17 and elucidate its mechanism of *Staphylococcus aureus* Cas9 (SauCas9) inhibition. AcrIIA17 adopts a previously uncharacterized protein fold and exists as a monomer in solution. Biochemical analyses reveal that AcrIIA17 inhibits SauCas9 activity in a strictly order-dependent manner, effectively suppressing DNA cleavage only when it engages Cas9 prior to single guide RNA (sgRNA) loading, whereas pre-assembled Cas9-sgRNA ribonucleoprotein (RNP) complexes are resistant to inhibition. Domain-mapping experiments demonstrate that AcrIIA17 directly binds to the bridge helix (BH) domain of SauCas9, and structure-guided mutagenesis confirms that this interaction is essential for its inhibitory function. Together, our findings identify AcrIIA17 as an Acr protein that targets the Cas9 BH domain and reveal the BH domain as a regulatory checkpoint in Cas9 activation.

## Highlights

• AcrIIA17 inhibits SauCas9 by preventing sgRNA loading
• AcrIIA17 directly binds the bridge helix region of SauCas9
• Inhibition requires AcrIIA17 binding before Cas9-sgRNA complex formation
• Structure-guided mutagenesis identifies residues critical for Cas9 inhibition

## Graphical abstract

**Figure:**
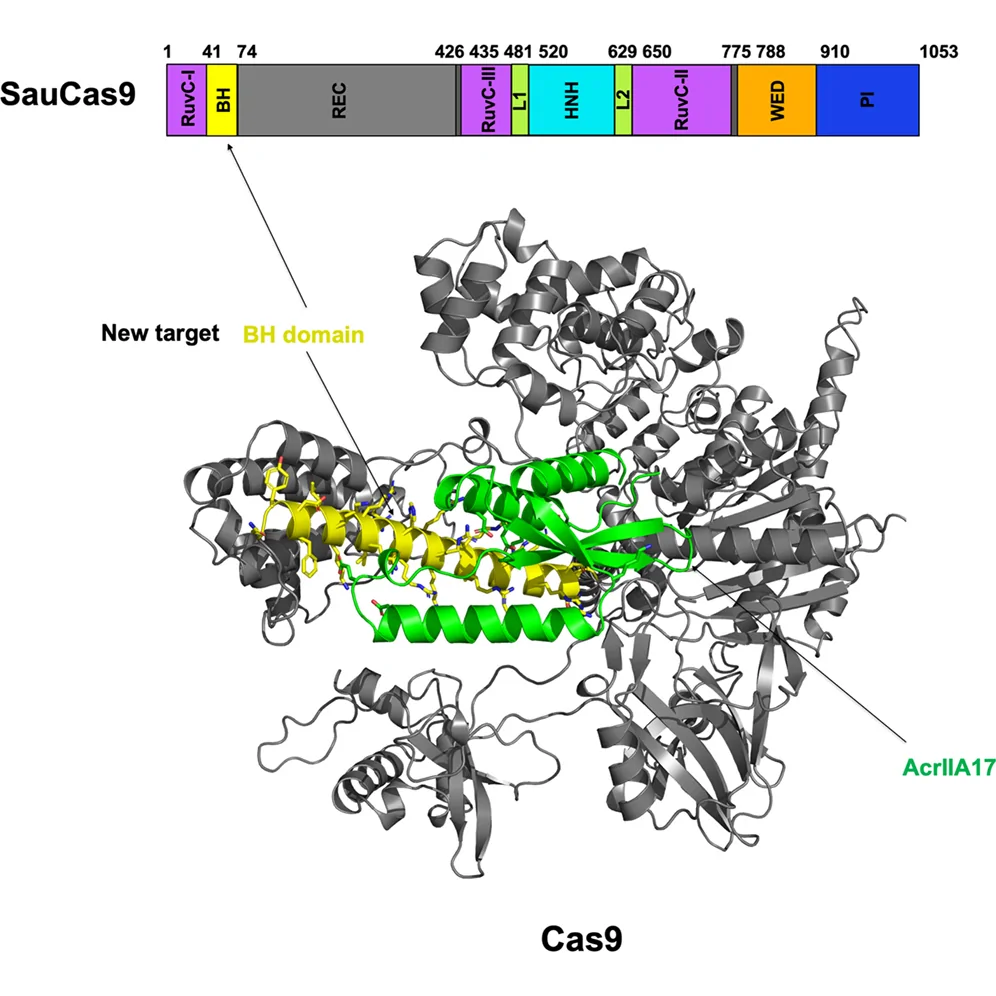

## Teaser

Molecular network; Neuroscience; Sensory neuroscience

## Introduction

Prokaryotes employ clustered regularly interspaced short palindromic repeat (CRISPR)-CRISPR-associated protein (Cas) systems as adaptive defense mechanisms that record prior encounters with foreign genetic elements and deploy RNA-guided nucleases to eliminate matching sequences upon reinfection.^1^^,^^2^^,^^3^ Among these systems, the type II-A CRISPR-Cas pathway from *Streptococcus pyogenes* has gained exceptional prominence as a genome-editing tool, owing to its minimal component requirement and efficient, programmable DNA cleavage.^4^^,^^5^

Cas9, the signature effector nuclease of the type II-A system, is a multidomain protein that undergoes a series of tightly regulated conformational transitions during ribonucleoprotein (RNP) assembly, target recognition, and DNA cleavage.^4^^,^^6^ In its inactive state, Cas9 exists in an apo conformation and must first associate with guide RNA (gRNA) to form an active RNP complex.^7^ This assembly step induces extensive rearrangements within the recognition (REC) lobe and nuclease (NUC) lobe, priming Cas9 for protospacer adjacent motif (PAM) recognition and subsequent target DNA interrogation.^8^ Upon target DNA binding, additional allosteric changes reposition the HNH and RuvC nuclease domains into catalytically competent conformations, ensuring precise and regulated DNA cleavage.^9^^,^^10^ These stepwise conformational transitions are essential for Cas9 function and represent critical regulatory checkpoints in the CRISPR-Cas immune response. A central structural element coordinating these conformational transitions is the bridge helix (BH) domain of Cas9. The BH domain connects RNA binding with catalytic activation by stabilizing single guide RNA (sgRNA) interactions and transmitting allosteric signals between the REC and NUC lobes.^11^^,^^12^ Previous structural and biochemical studies have shown that perturbation of the BH domain severely compromises sgRNA loading, RNP stability, and downstream DNA cleavage, underscoring its pivotal role in Cas9 activation.^12^ Despite its functional importance, relatively few regulatory proteins are known to directly target the BH domain to modulate Cas9 activity.

To evade CRISPR-mediated immunity, bacteriophages and mobile genetic elements have evolved small inhibitory proteins known as anti-CRISPR (Acr) proteins.^13^^,^^14^ Since their discovery, Acr proteins have emerged as a diverse class of natural Cas inhibitors that act through distinct molecular strategies, including blocking sgRNA loading,^15^ preventing DNA binding,^16^ or arresting the conformational transitions required for catalysis.^17^^,^^18^ Notably, many Acr proteins exert their inhibitory effects in a highly stage-specific manner, targeting discrete steps of the Cas9 functional cycle.^19^^,^^20^ Such temporal specificity allows Acr proteins to fine-tune Cas9 inhibition with remarkable precision.

AcrIIA17 is a representative type II-A Acr protein that has been identified in bacteriophages and plasmids and is known to suppress Cas9-mediated DNA cleavage.^21^ Despite its functional importance, the molecular basis underlying AcrIIA17-mediated inhibition has remained poorly understood. Although a recent study proposed the inhibition mechanism of AcrIIA17,^22^ the lack of structural information has hindered a mechanistic understanding of how AcrIIA17 recognizes its binding partner and how this interaction translates into effective Cas9 inhibition. In this study, we report the high-resolution three-dimensional structure of AcrIIA17 and elucidate its mechanism of Cas9 inhibition. Through biochemical assays, we demonstrate that AcrIIA17 inhibits *Staphylococcus aureus* Cas9 (SauCas9) activity in an order-dependent manner: AcrIIA17 effectively suppresses SauCas9 only when it engages Cas9 prior to sgRNA loading, whereas pre-assembled Cas9-sgRNA RNP complexes are resistant to inhibition. Structural and mutational analyses reveal that AcrIIA17 binds directly to the BH domain of SauCas9 and that this interaction is essential for its inhibitory activity. Structure-guided mutagenesis of AcrIIA17 and Cas9 confirms that disruption of the BH-binding interface abolishes inhibition, establishing the BH domain as the primary functional target of AcrIIA17. Together, our findings establish AcrIIA17 as an Acr protein that structurally targets the Cas9 BH domain and reveal the BH domain as a regulatory checkpoint in Cas9 activation.

## Results

### Structural determination of AcrIIA17 reveals a distinct and previously unreported fold

**Figure 1:**
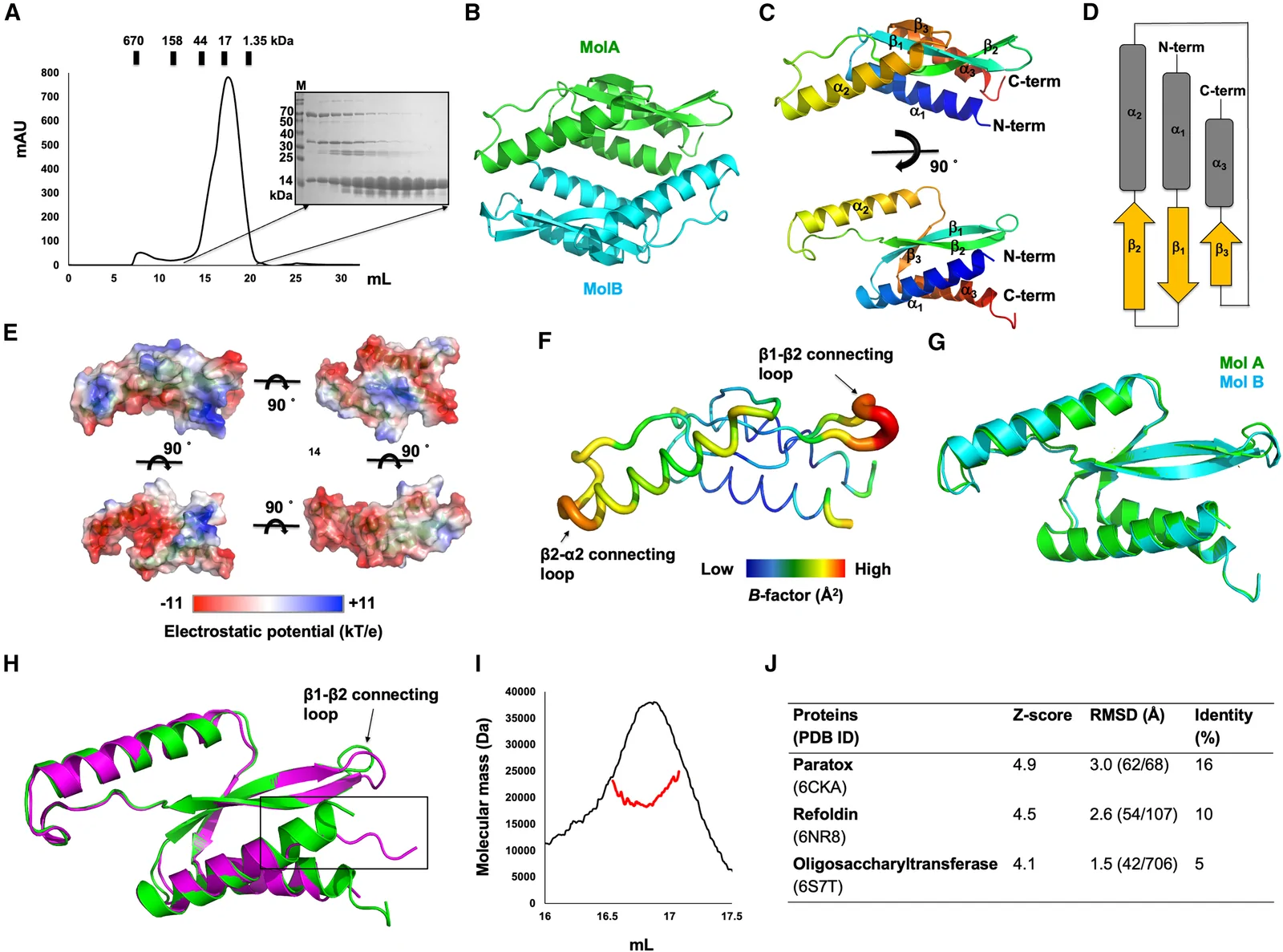
Overall architecture and structural features of AcrIIA17 (A) Size-exclusion chromatography (SEC) profile of purified AcrIIA17. SDS-PAGE analysis of fractions corresponding to the major SEC peak is shown right next to the main peak, with analyzed fractions indicated by black arrows. (B) Crystal packing of AcrIIA17 showing two independent molecules present in the asymmetric unit (ASU). (C) Ribbon representation of the AcrIIA17 structure, colored from the N terminus (blue) to the C terminus (red). Secondary structural elements are annotated. (D) Topology diagram illustrating the secondary structure arrangement of AcrIIA17. (E) Surface electrostatic potential map of AcrIIA17, displayed on a scale from −11.0 kT/e (red) to +11.0 kT/e (blue). (F) Distribution of *B*-factors mapped onto the AcrIIA17 structure and visualized in putty representation, with colors ranging from red (high) to violet (low). (G) Superposition of the two AcrIIA17 molecules within the ASU, demonstrating high structural similarity among independent copies. (H) Structural comparison between the experimentally determined AcrIIA17 structure (green) and the AF3-predicted model (magenta). Regions exhibiting notable deviations are highlighted by a black box. (I) SEC-MALS profile of AcrIIA17. The SEC elution trace is shown along the *x* axis, while the corresponding absolute molecular mass determined by MALS is plotted on the *y* axis. The experimental molecular mass derived from light-scattering measurements is indicated by the red curve overlaid on the chromatogram. (J) Summary of structural homology search results obtained using the DALI server.

To gain structural insight into the inhibitory mechanism of AcrIIA17, we recombinantly expressed and purified the full-length AcrIIA17 protein. Purification was achieved through immobilized Nickel-nitrilotriacetic acid (Ni-NTA) affinity chromatography, followed by size-exclusion chromatography (SEC). The SEC profile reproducibly displayed a single peak, and SDS-PAGE analysis confirmed that the eluted protein corresponded to AcrIIA17 with an apparent molecular mass of approximately 13.2 kDa (Figure 1A). The elution volume of the main SEC peak was intermediate between those of myoglobin (17 kDa) and vitamin B12 (1.35 kDa), suggesting that AcrIIA17 predominantly adopts a monomeric form in solution (Figure 1A). Protein fractions corresponding to the major SEC peak were subsequently used for crystallization screening, which yielded well-diffracting crystals. The crystal structure of AcrIIA17 was determined at 2.03 Å resolution by molecular replacement, using an AlphaFold3 (AF3)-predicted model as the search template.^23^ Comprehensive data collection and refinement statistics are summarized in Table 1.

**Table 1:** Data collection and refinement statistics

Data collection	
Space group	P2_1_2_1_2_1_
Unit cell parameter	–
a, b, c (Å)	45.08, 57.67, 81.19
αβ,γ (°)	90, 90, 90
Resolution range (Å)	28.83–2.03
Total reflections^a^	186,628 (19,125)
Unique reflection^a^	14,149 (1,369)
Multiplicity^a^	13.2 (14.0)
Completeness (%)^a^	99.44 (98.70)
Mean I/σ(I)^a^	18.06 (1.83)
R_merge_ (%)^a^^,^^b^	0.09514 (1.554)
Wilson B-factor (Å^b^)	39.14
Refinement	
Resolution range (Å)	28.83–2.03
Reflections^a^	14,137 (1,369)
R_work_^a^	0.2237 (0.2875)
R_free_^a^	0.2599 (0.3135)
No. of molecules in the asymmetric unit	1
No. of non-hydrogen atoms	1,821
Macromolecules	1,792
Solvent	29
Average B-factor values (Å^b^)	49.57
Macromolecules	49.66
Solvent	43.69
Ramachandran plot:	–
Favored/allowed/outliers (%)	99.02/0.49/0.49
Rotamer outliers (%)	0.00
Clashscore	5.83
RMSD bonds (Å)/angles (°)	0.009/1.17

a Values for the outermost resolution shell in parentheses.

b R_merge_ = Σ_h_ Σ_i_ |*I*(*h*)_i_ − <*I*(*h*)>|/Σ_*h*_ Σ_*i*_ I(*h*)_*i*_, where *I*(*h*) is the observed intensity of reflection *h*, and <*I*(*h*)> is the average intensity obtained from multiple measurements.

Crystals of AcrIIA17 belonged to the *P2*_*1*_*2*_*1*_*2*_*1*_ space group and contained two independent AcrIIA17 molecules within the asymmetric unit (ASU) (designated MolA and MolB; Figure 1B). The final refined model encompassed the complete protein sequence from residue M1 to F100 with extra LEHH residues which are derived from plasmid expression construct. Structurally, AcrIIA17 adopts a fold in which three β sheets form a central core that is surrounded by three α helices (Figures 1C and 1D). Surface electrostatic analysis revealed a relatively balanced distribution of positive and negative charges across the protein surface, providing no obvious indication of a strongly polarized interaction interface (Figure 1E). Analysis of atomic displacement parameters indicated modestly elevated *B*-factors at two loops, β1-β2 connecting loop and β2-α2 connecting loop, whereas the remainder of the structure exhibited uniformly low *B*-factor values (average 42.8 Å), consistent with a rigid and well-ordered fold (Figure 1F). Notably, the two AcrIIA17 molecules within the ASU adopted nearly identical conformations (Figure 1G).

Given the use of an AF3-generated model for molecular replacement, we next assessed the degree of agreement between the experimentally determined structure and the predicted model. Superposition of the two structures revealed strong overall correspondence, with minor deviations localized primarily to the β1-β2 connecting loop. The most structurally divergent region was the N-terminal segment, which was experimentally resolved as an α helix, whereas computational predictions suggested this region would adopt a loop conformation (Figure 1H).

Although many Acr proteins inhibit Cas9 as monomeric entities, accumulating evidence indicates that a subset of Acr proteins adopts higher oligomeric assemblies to exert their inhibitory functions.^15^^,^^24^ To assess the oligomeric state of AcrIIA17 in solution, we employed multi-angle light scattering (MALS) to directly determine the absolute molecular weight of AcrIIA17 in solution. MALS analysis revealed an average molecular mass of 16.8 kDa with a fitting error of 4.2% (Figure 1I). Given that the calculated molecular weight of monomeric AcrIIA17, including the C-terminal 6 × His tag, is approximately 13.2 kDa, the experimentally determined mass is consistent with a monomeric size. These results demonstrate that AcrIIA17 predominantly exists as a monomer in solution, providing a structural framework for its subsequent functional characterization.

To evaluate whether AcrIIA17 shares structural similarity with proteins of known function, we conducted a DALI-based structural homology search. The analysis identified paratox protein as the closest structural match (Figure 1J); however, the associated *Z* score (4.9) and low sequence identity (16%) indicated only marginal similarity. Other identified hits similarly exhibited low *Z* scores (4.1–4.5) and minimal sequence conservation (5%–10%), suggesting that AcrIIA17 adopts a unique structural architecture distinct from previously characterized protein folds. Collectively, these observations establish AcrIIA17 as a structurally distinct Acr protein.

### AcrIIA17 inhibits SauCas9 activity in a strictly order-dependent manner

**Figure 2:**
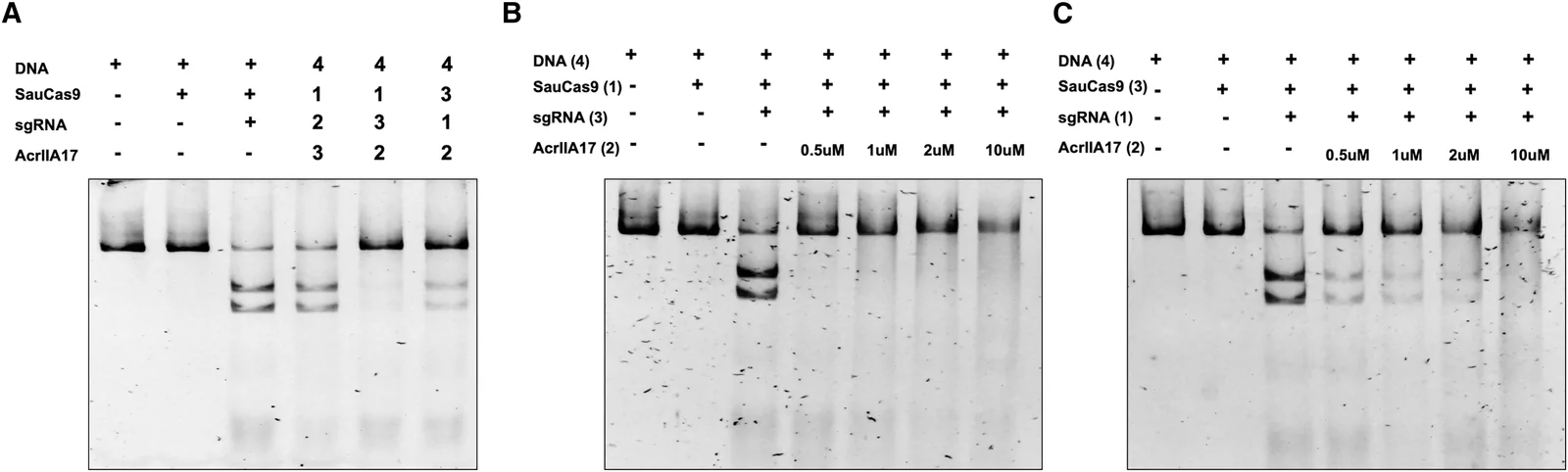
AcrIIA17 inhibits SauCas9-mediated DNA cleavage in an order-dependent manner (A) *In vitro* DNA cleavage assay illustrating the order-specific inhibitory effect of AcrIIA17 on SauCas9 activity. Numbers indicate the sequence in which reaction components were added. Reaction products were resolved on 4% polyacrylamide gels and visualized by SYBR GOLD staining. The presence (+) or absence (−) of individual components is indicated for each reaction condition. Experiments were repeated three times with comparable results. (B and C) Concentration-dependent inhibition assays performed under two distinct reaction schemes. In (B), SauCas9 (33 nM) was first mixed with AcrIIA17 prior to the addition of sgRNA. In (C), AcrIIA17 was pre-incubated with sgRNA (30 nM) before SauCas9 (33 nM) was introduced. In both cases, target DNA (2 nM) was added at the final step. The order of reagent addition is indicated by numbers next to each component. AcrIIA17 was added at final concentrations of 0.5, 1, 2.5, 5, and 10 μM, corresponding to the indicated lanes.

To characterize the inhibitory activity of AcrIIA17, we established an *in vitro* DNA cleavage assay using SauCas9, as AcrIIA17 exhibits broad-spectrum inhibitory activity against Cas9 proteins from diverse species.^21^ Cas9 is an RNA-guided endonuclease that requires assembly with an sgRNA to form a catalytically competent RNP complex. In this assay, SauCas9 was programmed with a synthetic sgRNA targeting a double-stranded DNA substrate containing a canonical 5′-NNGRR-3′ PAM sequence. Consistent with previous studies, co-incubation of SauCas9 and sgRNA resulted in efficient cleavage of the target DNA substrate, confirming proper RNP assembly and nuclease activation (Figure 2A). Interestingly, the inhibitory activity of AcrIIA17 was strongly dependent on the order of component addition. When AcrIIA17 was added after pre-assembly of the Cas9-sgRNA RNP complex, no appreciable reduction in DNA cleavage was observed (Figure 2A). Strikingly, however, pre-incubation of AcrIIA17 with Cas9 prior to sgRNA addition led to a pronounced suppression of Cas9-mediated DNA cleavage, indicating that AcrIIA17 selectively targets an early stage of Cas9 activation before RNP formation (Figure 2A).

**Figure 3:**
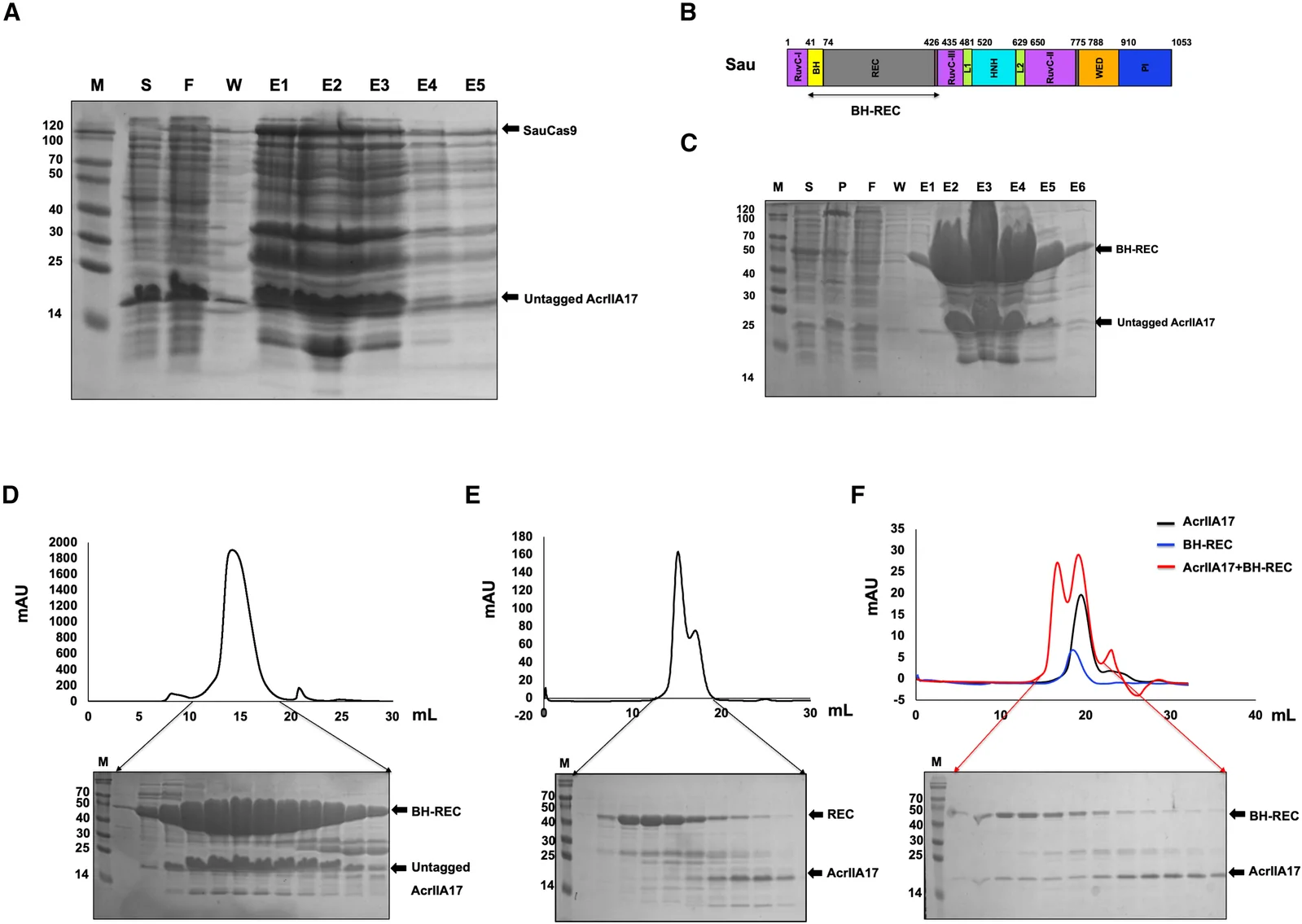
AcrIIA17 directly binds to Cas9 through the BH domain (A) Ni-NTA pull-down assay showing direct interaction between untagged AcrIIA17 and 6 × His-tagged SauCas9. Following incubation, protein complexes were captured using Ni-NTA resin and analyzed by SDS-PAGE with Coomassie brilliant blue staining. M, S, F, W, and E indicate marker, supernatant, flow-through, wash, and elution fractions, respectively. (B) Schematic representation of SauCas9 domain architecture. The BH-REC region that interacts with AcrIIA17 is indicated by an arrow. (C) Pull-down assay assessing the interaction between AcrIIA17 and BH-REC domain from SpyCas9. (D) SEC analysis of AcrIIA17 mixture with the BH-REC domain of SauCas9 obtained from pull-down assay at (C). Corresponding SDS-PAGE analysis is shown below the chromatogram. (E) SEC analysis of the protein mixture of AcrIIA17 and REC only domain from SauCas9. SDS-PAGE analysis of the indicated fractions is shown below. (F) SEC analysis of the purified protein mixture of AcrIIA17 and SauCas9 BH-REC. SDS-PAGE analysis of the indicated fractions is shown below.

To further examine the concentration dependence of this order-specific inhibition, we performed cleavage assays under two distinct conditions: (1) AcrIIA17 incubated with Cas9 prior to sgRNA loading (Figure 2B) and (2) AcrIIA17 incubated with sgRNA prior to Cas9 addition (Figure 2C). AcrIIA17 was tested over a concentration range of 0.5–10 μM. Under condition (1), Cas9 activity was fully suppressed even at a low concentration of 0.5 μM (Figure 3B). Under condition (2), AcrIIA17 inhibited Cas9 activity in a clear concentration-dependent manner, with near-complete suppression observed at a concentration of 10 μM (Figure 3C). These results demonstrate that AcrIIA17 inhibits Cas9 activity only when it engages Cas9 prior to sgRNA binding, revealing a strict order-dependent mode of inhibition that is mechanistically distinct from that of AcrIIA19.

### AcrIIA17 binds to SauCas9 through the BH domain

Acr proteins inhibit Cas9 activity through diverse molecular strategies, including sequestration of sgRNA, interference with target DNA binding, or direct engagement of Cas9 itself. Consistent with this diversity, several Acr proteins have been shown to interact with distinct structural elements of Cas9 to arrest its functional cycle.^18^^,^^25^^,^^26^^,^^27^^,^^28^^,^^29^^,^^30^ Given that the inhibitory activity of AcrIIA17 is strictly dependent on reaction order, we hypothesized that AcrIIA17 directly associates with Cas9 at a stage preceding RNP assembly.

To test this hypothesis, we first examined whether AcrIIA17 directly binds to full-length SauCas9 using a Ni-NTA pull-down assay. In this assay, untagged AcrIIA17 was co-eluted with 6 × His-tagged SauCas9, providing evidence for a direct association between the two proteins (Figure 3A). To delineate the Cas9 region responsible for AcrIIA17 binding, we next performed pull-down assays using individually expressed domains of SauCas9. SauCas9 consists of multiple functional modules, including the BH, REC lobe, HNH nuclease domain, wedge (WED) domain, and PAM-interacting (PI) domain (Figure 3B). A series of domain constructs were generated and tested for interaction with AcrIIA17 by pull-down assays. These experiments revealed that AcrIIA17 did not associate detectably with the REC or HNH domains. In contrast, AcrIIA17 was robustly pulled down by constructs containing the BH-REC domain, indicating a strong and specific interaction (Figure 3C). To further substantiate this domain specificity, we performed SEC analyses using protein mixtures obtained from pull-down assay. Clear co-migration was observed when AcrIIA17 was mixed with BH-REC protein, confirming their direct binding in solution (Figure 3D). These results establish the BH-REC domain as the primary binding site for AcrIIA17 on Cas9.

To further determine whether AcrIIA17 binds to the BH domain or the REC domain of Cas9, we attempted to generate expression constructs encoding the isolated BH and REC domains. However, the BH domain consists of only ∼30 amino acids, rendering it unsuitable for independent expression and purification. As an alternative approach, we expressed a SauCas9 construct lacking the BH domain, corresponding to the REC domain alone, and assessed its interaction with AcrIIA17. When mixed with AcrIIA17 and analyzed by SEC, the two proteins eluted as separate peaks, and no co-migration was observed by SDS-PAGE analysis, indicating a lack of interaction (Figure 3F). Taken together, these results indicate that AcrIIA17 does not bind to the REC domain but instead interacts with the BH domain, thereby inhibiting SauCas9 activity.

Finally, to further validate the interaction between AcrIIA17 and the BH-REC region of SauCas9, we performed SEC using purified proteins. Purified AcrIIA17 and the BH-REC construct were mixed and incubated prior to SEC analysis. Whereas AcrIIA17 and the BH-REC construct eluted at their respective positions when analyzed individually, the protein mixture produced a distinct peak that eluted earlier than either protein alone, consistent with the formation of a higher-molecular-weight complex (Figure 3F). SDS-PAGE analysis of the corresponding fractions confirmed the presence of both AcrIIA17 and the BH-REC construct within the same peak. These results provide direct biochemical evidence that AcrIIA17 interacts with the BH-REC region of SauCas9 and further support the interaction identified by pull-down assays.

### Direct binding of AcrIIA17 to the Cas9 BH domain is critical for its inhibitory function

**Figure 4:**
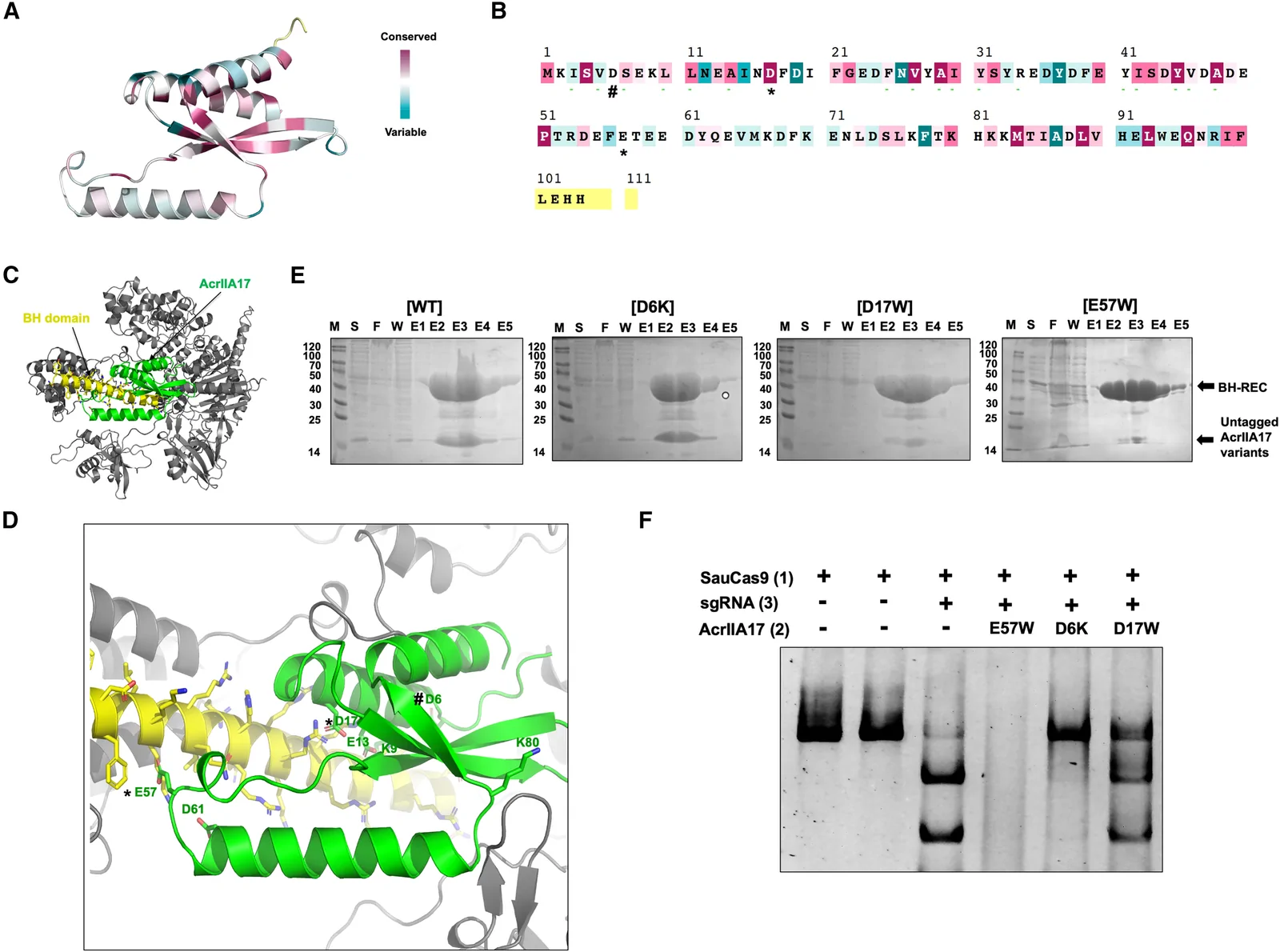
BH domain binding is essential for AcrIIA17-mediated inhibition (A) Cartoon representation of AcrIIA17 mapped with sequence conservation scores calculated by ConSurf. (B) Primary amino acid sequence of AcrIIA17 colored according to ConSurf-derived conservation values. Asterisks (∗) denote residues mutated to disrupt AcrIIA17-SauCas9 binding, while hash symbol (#) indicates residue used as negative controls. (C) Complex structural model generated by AF3 for SauCas9. In the model, the SauCas9 structure is shown in gray, AcrIIA17 is shown in green, and the Cas9 BH domain is highlighted in yellow. The ipTM and pLDDT values for the AcrIIA17-SauCas9 complex model were 0.89 and 88.57, respectively. (D) Close-up view of AcrIIA17 bound to the BH domain in the complex model. Asterisks (∗) indicate residues predicted to be critical for disrupting the interaction between AcrIIA17 and SauCas9 and were therefore subjected to mutagenesis, whereas hash symbols (#) denote residues predicted not to be involved in binding and used as negative controls. (E) Pull-down assays examining the interaction of Cas9 BH-REC domain with AcrIIA17 or its variants. M, S, F, W, and E indicate marker, supernatant, flow-through, wash, and elution fractions, respectively. (F) *In vitro* DNA cleavage assay demonstrating inhibition of SauCas9 activity by AcrIIA17 or its variants. Reaction products were resolved on 4% polyacrylamide gels and visualized by SYBR GOLD staining. Numbers indicate the order of component addition. + and − denote presence or absence of individual reagents.

Given that AcrIIA17 inhibits Cas9 through binding to the BH domain, the subsequent question was to elucidate the molecular mechanism underlying this interaction. To investigate how AcrIIA17 recognizes and binds to the BH domain of SauCas9, we first identified functionally important and evolutionarily conserved residues within AcrIIA17 using the ConSurf server.^31^ This analysis showed that conserved residues that might be important for Cas9 interaction, are primarily located in the central core domain composed of three β sheets (Figures 4A and 4B). Next, we performed structural modeling of the AcrIIA17-SauCas9 complex using AF3 to predict how AcrIIA17 binds to the BH domain. The interfacial predicted TM-score (ipTM) and predicted Local Distance Difference Test (pLDDT) values for the AcrIIA17-SauCas9 complex model were 0.89 and 88.57, respectively (Figure S1). Notably, modeling analyses revealed that AcrIIA17 preferentially associates with the BH domain of Cas9, despite the absence of any predefined binding site constraints (Figure 4C). AcrIIA17 was found to engage the α-helical BH domain in a stable manner. In particular, the central core region of AcrIIA17, which is enriched in conserved residues identified by ConSurf analysis, forms a wrapping interface around the BH domain (Figure 4D). This observation indicates a strong correspondence between residues predicted to be functionally important in AcrIIA17 and those directly involved in Cas9 binding, supporting the functional relevance of this interaction. The proposed interface is based on computational modeling. The precise molecular details of the interaction will require future experimental validation by high-resolution structural studies of the AcrIIA17-SauCas9 complex.

Based on these sequence and structure analyses, we selected two residues—D17 and E57—located at the predicted Cas9-interacting surface of AcrIIA17 for mutational analysis (Figure 4D). D17 was selected as a representative highly conserved residue according to sequence analyses, while E57 was chosen as a representative non-conserved residue (Figure 4B). To disrupt potential electrostatic and steric interactions, each residue was substituted with tryptophan, generating the mutants D17W and E57W. A D6W mutant, predicted not to participate in binding, was also generated and used as a negative control. To assess the impact of these mutations on Cas9 binding, we performed pull-down assays. Wild-type AcrIIA17 and negative control D6W exhibited robust binding to BH-Cas9, whereas D17W and E57W displayed markedly reduced or abolished interaction (Figure 4E).

We next examined whether the loss of SauCas9 binding correlated with functional inhibition. *In vitro* DNA cleavage assays demonstrated that mutant defective in SauCas9 binding, D17W, partially failed to suppress Cas9-mediated DNA cleavage (Figure 4F). In contrast, the mutant retaining binding activity (D6K) exhibited correspondingly detectable inhibitory effects. These results establish a strong correlation between BH-domain binding and Cas9 inhibition by AcrIIA17. For reasons that remain unclear, the E57W mutant, which failed to bind Cas9, consistently exhibited disappearance of the DNA bands accompanied by severe smearing. We speculate that this behavior results from reduced solubility of the E57W mutant caused by the mutation. To investigate this possibility, we compared the biochemical properties of the wild-type and mutant proteins during purification. Based on SEC behavior (Figure S2) and the purity of the purified proteins (Figure S3A), we did not observe any significant differences between the wild-type and mutant proteins. All mutants were expressed and purified with similar yields and exhibited SEC profiles comparable to that of the wild-type protein. We further evaluated the structural integrity of the purified proteins using circular dichroism (CD) spectroscopy. The CD spectra of all mutants closely resembled that of the wild-type AcrIIA17 (Figure S3B), indicating that their overall secondary structures were preserved. Taken together, these results suggest that the mutations do not cause substantial changes in protein solubility, folding, or structural stability. Therefore, the observed phenotype is unlikely to result from mutation-induced protein misfolding or loss of solubility.

Collectively, these structure-guided mutagenesis experiments demonstrate that AcrIIA17 inhibits SauCas9 through a direct interaction with the BH domain and that this interaction critically depends on a set of conserved, surface-exposed residues on AcrIIA17. Disruption of this interface abolishes both Cas9 binding and inhibitory activity, providing definitive evidence that BH-domain engagement is central to the mechanism of AcrIIA17-mediated SauCas9 inhibition.

## Discussion

In this study, we provide a structural and mechanistic characterization of AcrIIA17 and show that it inhibits Cas9 through a mode distinct from those reported for other Acr proteins. By combining high-resolution crystallography, biochemical assays, domain-mapping experiments, and structure-guided mutagenesis, we demonstrate that AcrIIA17 inhibits SauCas9 by directly targeting the BH domain. These findings establish the BH domain as a vulnerable regulatory node for Acr-mediated control of Cas9 activity.

Most Acr proteins characterized to date inhibit Cas9 by interfering with sgRNA binding, target DNA recognition, or nuclease activation through engagement of domains such as the REC lobe, WED domain, or catalytic HNH and RuvC domains. In contrast, AcrIIA17 selectively targets the BH domain, a short but functionally critical α-helical element that couples sgRNA binding to global conformational rearrangements required for Cas9 activation. Although the importance of the BH domain for RNP assembly and catalytic competence has been well documented,^11^^,^^12^ direct targeting of this region by an Acr protein has not previously been demonstrated with experimentally determined structural information. A previous study by Wang et al.^22^ proposed that AcrIIA17 inhibits SauCas9 through interaction with the REC lobe and consequent interference with DNA recognition. While our findings support the overall conclusion that AcrIIA17 suppresses SauCas9 activity, our structural and biochemical analyses further localize the primary interaction site to the BH domain. Because the BH domain is functionally and structurally integrated with the REC lobe and plays an essential role in sgRNA loading and Cas9 activation, perturbation of this region may account for the inhibitory effects previously attributed to the REC lobe. Our data therefore refine the mechanistic model of AcrIIA17 inhibition by identifying a specific structural target and suggesting that inhibition occurs primarily at the stage of RNP assembly rather than target DNA binding.

A defining feature of AcrIIA17 is its strict dependence on reaction order. Our biochemical assays demonstrate that AcrIIA17 potently inhibits SauCas9 only when it encounters Cas9 prior to sgRNA loading, whereas pre-assembled Cas9-sgRNA RNP complexes are largely resistant to inhibition. This behavior can be readily rationalized by our binding and modeling data. The BH domain is solvent-exposed and accessible in the apo form of Cas9, enabling AcrIIA17 binding. Upon sgRNA association, however, the BH domain becomes buried within the RNP complex and adopts a conformation stabilized by extensive RNA-protein interactions. In this RNP-bound state, the BH domain is no longer accessible to AcrIIA17, rendering inhibition ineffective. The BH domain occupies a central position at the interface between the REC and NUC lobes of Cas9 and undergoes substantial rearrangement upon sgRNA binding.^11^^,^^12^ This strategic location likely explains why BH engagement by AcrIIA17 is sufficient to prevent RNP formation while having little effect once the RNP complex has already assembled. This mechanism places AcrIIA17 among a subset of Acr proteins that act at a very early checkpoint in the Cas9 activation pathway. Such early-stage inhibition may be particularly advantageous for mobile genetic elements, as it prevents the formation of an active surveillance complex rather than attempting to disable an already functional nuclease. From a regulatory perspective, BH targeting provides a highly specific means of controlling Cas9 activity without directly interfering with DNA binding or cleavage chemistry. Although our biochemical and mutational analyses identify the BH domain as the primary determinant of AcrIIA17 recognition, the interaction studies were necessarily performed using a larger BH-REC construct because the isolated BH domain is not amenable to independent expression and purification. Therefore, we cannot completely exclude the possibility that adjacent regions within the REC lobe contribute to complex formation or stabilize the interaction. A high-resolution structure of the AcrIIA17/Cas9 complex will ultimately be required to define the complete binding epitope and molecular contacts at atomic detail.

The crystal structure of AcrIIA17 reveals a compact fold composed of a three-stranded β sheet core flanked by α helices, representing a structural architecture distinct from those of previously characterized Acr proteins. Structure-based conservation analysis indicates that residues critical for Cas9 interaction cluster within the central core of AcrIIA17. Consistent with this observation, AF3-based modeling predicts that this conserved surface wraps around the α-helical BH domain of Cas9, forming a complementary interface. Structure-guided mutagenesis further validates this model. Mutation of conserved residues such as D17 significantly impaired BH binding and abolished Cas9 inhibition, whereas mutations at positions not predicted to contact Cas9 had little effect. The strong correlation between BH binding affinity and inhibitory potency establishes a direct causal relationship between AcrIIA17-BH interaction and functional inhibition. These results also suggest that even subtle perturbations of the BH-binding interface can dramatically affect AcrIIA17 activity, highlighting the precision with which Acr proteins exploit key structural features of Cas9. Although our biochemical, structural, and mutational analyses strongly support a model in which AcrIIA17 inhibits SauCas9 by binding the BH domain and preventing sgRNA loading, the present study did not directly evaluate whether AcrIIA17 can interact with or modify free sgRNA. Therefore, we cannot formally exclude additional effects on sgRNA, although the available evidence favors a Cas9-directed inhibitory mechanism.

Beyond its biological significance, the discovery of BH-targeting inhibition has important implications for the rational control of CRISPR-Cas9 systems in biotechnology and therapeutic applications. Because BH engagement selectively blocks RNP assembly without directly perturbing DNA binding or cleavage once the RNP is formed, AcrIIA17 or engineered derivatives could be used as temporal regulators of Cas9 activity. Such inhibitors may be particularly useful for reducing off-target effects by limiting the window during which active RNP complexes can form. Moreover, the structural insights provided here may inform the design of small molecules or peptides that mimic AcrIIA17 and selectively target the BH domain. Given the central role of the BH domain in Cas9 activation, such agents could serve as general Cas9 modulators applicable across multiple orthologs.

In summary, our study establishes AcrIIA17 as a structurally distinct Acr protein that inhibits SauCas9 by directly targeting the BH domain. This interaction blocks RNP assembly in a strictly order-dependent manner. These findings expand the known repertoire of Acr mechanisms, highlight the BH domain as a critical vulnerability in Cas9, and provide a framework for developing strategies to precisely control CRISPR-based genome-editing technologies.

### Limitations of the study

This study has several limitations. First, although biochemical analyses and structure-guided mutagenesis support the interaction of AcrIIA17 with the BH domain of SauCas9, a high-resolution experimental structure of the AcrIIA17-SauCas9 complex was not obtained. Therefore, the precise molecular interface and binding stoichiometry remain to be experimentally established. Second, the proposed binding mode is based partly on AF3 modeling and should be interpreted as a working model rather than a definitive complex structure. Finally, because the mechanistic analyses were performed predominantly with SauCas9, it remains unclear whether AcrIIA17 can recognize and inhibit other Cas9 orthologs through a similar BH-targeting mechanism.

## Resource availability

### Lead contact

Further information and requests for resources should be directed to and will be fulfilled by the lead contact, Hyun Ho Park (xrayleox@cau.ac.kr).

### Materials availability

Plasmids and other materials generated in this study are available from the lead contact upon reasonable request.

### Data and code availability

• The atomic coordinates and structure factors for the AcrIIA17 crystal structure have been deposited in the Protein Data Bank and are publicly available under accession code (23AA). The accession code is also provided in the key resources table. All other data supporting the findings of this study are available within the paper and its supplemental information. Uncropped blot images are provided in Figure S4.
• This study did not generate original code. The software and computational tools used in this study are publicly available and are listed in the key resources table.
• Any additional information required to reanalyze the data reported in this paper is available from the lead contact upon reasonable request.

## Acknowledgments

We would like to thank the 5C beamline staff of the Pohang Accelerator Laboratory (Pohang, Korea) for their assistance during data collection. This study was supported by a 10.13039/501100003725National Research Foundation of Korea (NRF) grant funded by the Korean government (10.13039/501100014188MSIT) (RS-2025-02316334 and RS-2026-25470081).

## Author contributions

H.H.P. designed the research and supervised the project. G.E.K. and H.B.J. expressed and purified the proteins and performed crystallization and structure determination. G.E.K. and H.B.J. conducted biochemical and EMSA experiments. G.E.K. and Y.J.K. performed structural prediction and MALS analysis. H.H.P., G.E.K., and H.B.J. interpreted the data and wrote the manuscript with contributions from all authors. All authors reviewed and approved the final manuscript.

## Declaration of interests

The authors declare no competing interests.

## STAR★Methods

### Key resources table

**Table:** 

REAGENT or RESOURCE	SOURCE	IDENTIFIER
**Bacterial and virus strains**		

*Escherichia coli* BL21(DE3)	Novagen	Cat# 69450
*Escherichia coli* (DH5α)	Thermo Fisher Scientific	Cat# EC0112

**Chemicals, peptides, and recombinant proteins**		

Isopropyl β-D-1-thiogalactopyranoside (IPTG)	Sigma-Aldrich	Cat# I6758
Imidazole	Sigma-Aldrich	Cat# I5513
Sodium chloride	Merck	Cat# S3014
Dithiothreitol (DTT)	Thermo Fisher Scientific	Cat# D9779

**Deposited data**		

AcrIIA17 crystal structure	This paper	PDB: 23AA

**Recombinant DNA**		

acrIIA17 gene (GenBank accession no. WP_074626943.1)	synthesized	N/A
SauCas9 gene	addgene	#101086
AcrIIA17 expression plasmid	This paper	N/A
SauCas9 expression plasmid	This paper	N/A

**Software and algorithms**		

AlphaFold3	Google DeepMind and Isomorphic Labs	https://alphafoldserver.com/
PyMOL	Schrödinger, LLC	RRID: SCR_000305
PHENIX	McCoy et al.^32^	RRID: SCR_014224
COOT	Emsley^33^	RRID: SCR_014222

### Experimental model and study participant details

Escherichia coli [DH5α] was used for plasmid construction and propagation. Cells were cultured in Luria–Bertani (LB) medium. Escherichia coli [BL21(DE3)] was used for recombinant protein expression.

The bacterial strains used in this study are listed in the Key Resources Table. No animals, human participants or samples, plants, primary cell cultures, or eukaryotic cell lines were used in this study. Therefore, information concerning age, developmental stage, sex, gender, ancestry, race, ethnicity, cell-line authentication, mycoplasma testing, and institutional oversight for animal or human research is not applicable. Because the experimental models were bacterial strains and purified recombinant proteins, the influence of sex or gender on the study outcomes could not be assessed and is not applicable to the conclusions of this study.

### Method details

#### Cloning, overexpression, and purification of AcrIIA17 and its variants, Cas9, and individual Cas9 domains

The *acrIIA17* gene encoding residues 1–100 from *Streptococcus gallolyticus* (GenBank accession no. WP_074626943.1) was chemically synthesized (Bionics, Daejeon, Republic of Korea) and subcloned into the pET21a expression vector (Novagen) via the *NdeI* and *XhoI* restriction sites. The resulting construct was transformed into *Escherichia coli* BL21(DE3) competent cells for recombinant protein expression. Transformed cells were grown in 1 L of lysogeny broth (LB) supplemented with 50 μg/mL ampicillin at 37°C. Upon reaching an OD_600_ of approximately 0.8, cultures were shifted to 20°C and protein expression was induced by the addition of 0.5 mM isopropyl β-D-1-thiogalactopyranoside (IPTG). Cells were incubated for an additional 20 h at 20°C prior to harvesting. Cell pellets were collected by centrifugation at 2,000 × g for 15 min at 4°C and resuspended in 25 mL of lysis buffer containing 20 mM Tris–HCl (pH 8.0) and 500 mM NaCl. Cell disruption was achieved by sonication, and insoluble debris was removed by centrifugation at 10,000 × g for 30 min at 4°C. The clarified supernatant was incubated with Ni–NTA agarose resin for 3 h at 4°C to allow affinity binding of the His-tagged protein. Following incubation, the resin was transferred to a gravity-flow column (Bio-Rad) and washed extensively with 100 mL lysis buffer to remove non-specifically bound proteins. AcrIIA17 was subsequently eluted using an imidazole-containing buffer (20 mM Tris–HCl, pH 8.0, 500 mM NaCl, and 250 mM imidazole). Further purification was performed by size-exclusion chromatography (SEC) on a Superdex 200 10/300 GL column (GE Healthcare) connected to an ÄKTA Explorer system and equilibrated in SEC buffer consisting of 20 mM Tris–HCl (pH 8.0) and 150 mM NaCl. Fractions corresponding to AcrIIA17 were pooled and concentrated to 13.4 mg/mL for crystallization trials. Protein purity was assessed by SDS–PAGE.

Expression plasmids encoding full-length *Staphylococcus aureus* Cas9 (SauCas9: #101086) were obtained from Addgene. To generate constructs encoding SauBH–REC of SauCas9, the corresponding regions were amplified by PCR from SauCas9 templates. PCR products were cloned into the pET24a vector using *NdeI* and *XhoI* restriction sites. Recombinant Cas9 domain proteins were expressed and purified following the same protocol described for AcrIIA17, with the exception that kanamycin was used as the selection antibiotic.

#### Crystallization and X-ray diffraction data collection

Crystallization of AcrIIA17 was carried out at 20°C using the hanging-drop vapor diffusion technique. Crystallization drops were prepared by combining equal volumes (1 μL each) of purified AcrIIA17 protein solution (13.4 mg/mL in SEC buffer) and reservoir solution composed of 0.1 M Tris–HCl (pH 8.5), 16% (w/v) PEG 8000, 14% (w/v) PEG 400, and 0.1 M magnesium chloride (MgCl_2_). The mixed drops were equilibrated against 500 μL of reservoir solution. Well-formed crystals suitable for diffraction analysis appeared after extended incubation, with optimal crystals observed after approximately 99 days.

For cryogenic data collection, individual crystals were transferred to a cryoprotectant solution consisting of the reservoir solution supplemented with 40% (v/v) glycerol prior to flash-cooling. X-ray diffraction data were collected at −178°C on beamline BL-5C at the Pohang Accelerator Laboratory (PAL; Pohang, Republic of Korea). Diffraction images were indexed, integrated, and scaled using the HKL2000 software package to generate processed datasets for subsequent structural analysis.^34^

#### Structure determination and refinement

The crystal structure of AcrIIA17 was determined by molecular replacement using the PHASER algorithm implemented in the PHENIX software package.^32^ A predicted structural model generated by AlphaFold3 (AF3) served as the search template, which enabled unambiguous phase determination. Following successful phasing, an initial atomic model was constructed using the automated AutoBuild workflow in PHENIX. Iterative cycles of manual model correction and refinement were subsequently performed using Coot^33^ and phenix.refine^35^ to improve model geometry and agreement with the experimental data. The quality of the final refined model was assessed using MolProbity,^36^ ensuring appropriate stereochemistry and overall structural reliability. Molecular graphics and structural representations were generated using PyMOL.^37^

#### Multi-angle light scattering (MALS) analysis

The oligomeric state and absolute molecular mass of AcrIIA17 in solution were determined by SEC-coupled to multi-angle light scattering (SEC–MALS). Purified protein samples were loaded onto a Superdex 200 Increase 10/300 GL (24 mL) column (GE Healthcare) that had been equilibrated with SEC buffer, and chromatographic separation was performed at a constant flow rate of 0.5 mL/min. SEC–MALS measurements were carried out at 20°C using a DAWN TREOS multi-angle light scattering detector (Wyatt Technology, Santa Barbara, USA) integrated with an ÄKTA Explorer chromatography system. Bovine serum albumin was analyzed under identical conditions as a calibration reference to validate the accuracy of molecular weight determination. Light-scattering data were collected and analyzed using ASTRA software, which was used to calculate absolute molecular masses and assess the molecular weight distribution of the proteins.

#### *In vitro* anti-CRISPR activity assay

The inhibitory activity of AcrIIA17 and its mutant derivatives against SauCas9-mediated DNA cleavage was assessed using an *in vitro* target cleavage assay. Recombinant SauCas9 enzymes (New England Biolabs) were assembled with *in vitro*–transcribed single guide RNA (sgRNA), which was generated using the HiScribe T7 Quick High Yield RNA Synthesis Kit (NEB). Cas9 proteins were incubated with sgRNA in NEBuffer r3.1 (NEB) at 20°C for 15 min to allow formation of ribonucleoprotein (RNP) complexes.

Purified wild-type AcrIIA17 or mutant proteins were subsequently added to the SauCas9–sgRNA mixtures and incubated for an additional 15 min at 20°C. Target DNA substrates were then introduced, and cleavage reactions were carried out at 37°C for 30 min. Enzymatic reactions were terminated by the addition of Proteinase K, followed by incubation for 10 min to degrade proteins. Reaction products were resolved on 4% polyacrylamide gels, and DNA fragments were visualized by SYBR GOLD staining. Gel images were analyzed to quantify DNA cleavage efficiency and to evaluate the inhibitory effects of wild-type AcrIIA17 and its mutant variants on Cas9-catalyzed target DNA cleavage.

#### SEC assay for analyzing complex formation

Complex formation between AcrIIA17, AcrIIA17 variants, and SauCas9, as well as interactions with individual domain of SauCas9, was evaluated by SEC. Each purified AcrIIA17 and AcrIIA17 variants were combined with full-length SauCas9 protein or isolated Cas9 domain and incubated at 4°C for 1 h to allow complex assembly.

Following incubation, the protein mixtures were loaded onto a Superdex 200 Increase 10/300 GL column (GE Healthcare) equilibrated with SEC buffer consisting of 20 mM Tris–HCl (pH 8.0) and 150 mM NaCl. Fractions corresponding to elution peaks were collected and subjected to SDS–PAGE analysis, with proteins visualized by Coomassie Brilliant Blue staining. Complex formation was assessed by examining shifts in elution profiles and co-elution of AcrIIA17 (or its mutant variants) with Cas9 proteins or their respective domains.

#### Pulldown assay for analyzing complex formation

Protein–protein interactions between AcrIIA17 and full-length SauCas9, as well as interactions involving individual Cas9 domains and AcrIIA17 mutant variants, were examined using a Ni–NTA–based pull-down assay. Cell lysates containing recombinant AcrIIA17 were combined with lysates expressing Cas9 proteins or isolated Cas9 domains and incubated at 4°C for 1 h to allow complex formation. In parallel, lysates containing AcrIIA17 mutant proteins were mixed with corresponding Cas9 or Cas9 domain lysates under identical conditions. Following incubation, the protein mixtures were supplemented with Ni–NTA agarose resin and gently agitated at 4°C for an additional 2 h to capture His-tagged complexes. The resin-bound proteins were transferred to a gravity-flow column (Bio-Rad, Hercules, CA, USA) and extensively washed with lysis buffer to remove nonspecifically associated proteins. Bound proteins were then eluted using 3 mL of elution buffer containing 20 mM Tris–HCl (pH 8.0), 500 mM NaCl, and 250 mM imidazole. Eluted fractions were analyzed by SDS–PAGE followed by Coomassie Brilliant Blue staining. Interaction between AcrIIA17 (or its mutant forms) and Cas9 proteins or their individual domains was evaluated based on the presence and co-elution of corresponding protein bands.

#### Structural modeling of the AcrIIA17–SauCas9 complexe

Computational docking was performed to generate structural models of AcrIIA17 in complex with SauCas9. Docking calculations were carried out using AF3.^23^ Amino acid sequences of SauCas9 and AcrIIA17 were provided as input for complex structure prediction. All remaining settings, including random initialization parameters and model configuration options, were assigned automatically by the prediction algorithm. From the ensemble of generated models, the highest-confidence structure was selected based on composite AlphaFold confidence metrics, including pLDDT, interfacial predicted TM-score (ipTM), and predicted TM-score (pTM). The selected complex model was subsequently visualized and examined using PyMOL.

#### Mutagenesis

Site-directed mutations were introduced using a PCR-based QuickChange mutagenesis strategy (Stratagene, San Diego, CA, USA) in accordance with the manufacturer’s instructions. All nucleotide substitutions were verified by DNA sequencing to ensure the accuracy of the introduced mutations. Recombinant mutant proteins were expressed and purified using the same procedures as those applied for the wild-type AcrIIA17 protein.

### Quantification and statistical analysis

All biochemical experiments were performed in two independent experiments, and representative results are shown. The independent replicate data, including the original and uncropped blot and gel images, are provided in Figure S4 where applicable. No statistical method was used to predetermine sample size. No randomization or blinding was performed because the study involved purified recombinant proteins and *in vitro* biochemical assays rather than animals or human participants. No experimental data were excluded from the analyses.
