# Supplementary material for: Structural insights into Cas9 inhibition by AcrIIA17 via bridge helix interaction
Source: iScience. 2026 Aug 19;29(9):117218. doi: 10.1016/j.isci.2026.117218 (PMC13520725; doi:10.1016/j.isci.2026.117218)
Supplement: Supplementary file 1 [file mmc1.pdf]

iScience, Volume *29*

Supplemental information

Structural insights into Cas9 inhibition by AcrIIA17 via bridge helix interaction

Gi Eob Kim, Hyo Been Jin, Yong Jun Kang, and Hyun Ho Park

Structural insights into Cas9 inhibition by AcrIIA17 via bridge helix interaction

Gi Eob Kim[1,2,#], Hyo Been Jin[1,2,#], Yong Jun Kang[1,2], and Hyun Ho Park[1,2,*]

[1]College of Pharmacy, Chung-Ang University, Seoul 06974, Republic of Korea

[2]Department of Global Innovative Drugs, Graduate School of Chung-Ang University, Seoul 06974, Republic of Korea

[#]These authors contributed equally to the work

*Correspondence to:
Hyun Ho Park; College of Pharmacy, Chung-Ang University, Seoul 06974, Republic of Korea; Tel: +82-2-820-5930; Fax: +82-2-820-3033; Email: xrayleox@cau.ac.kr

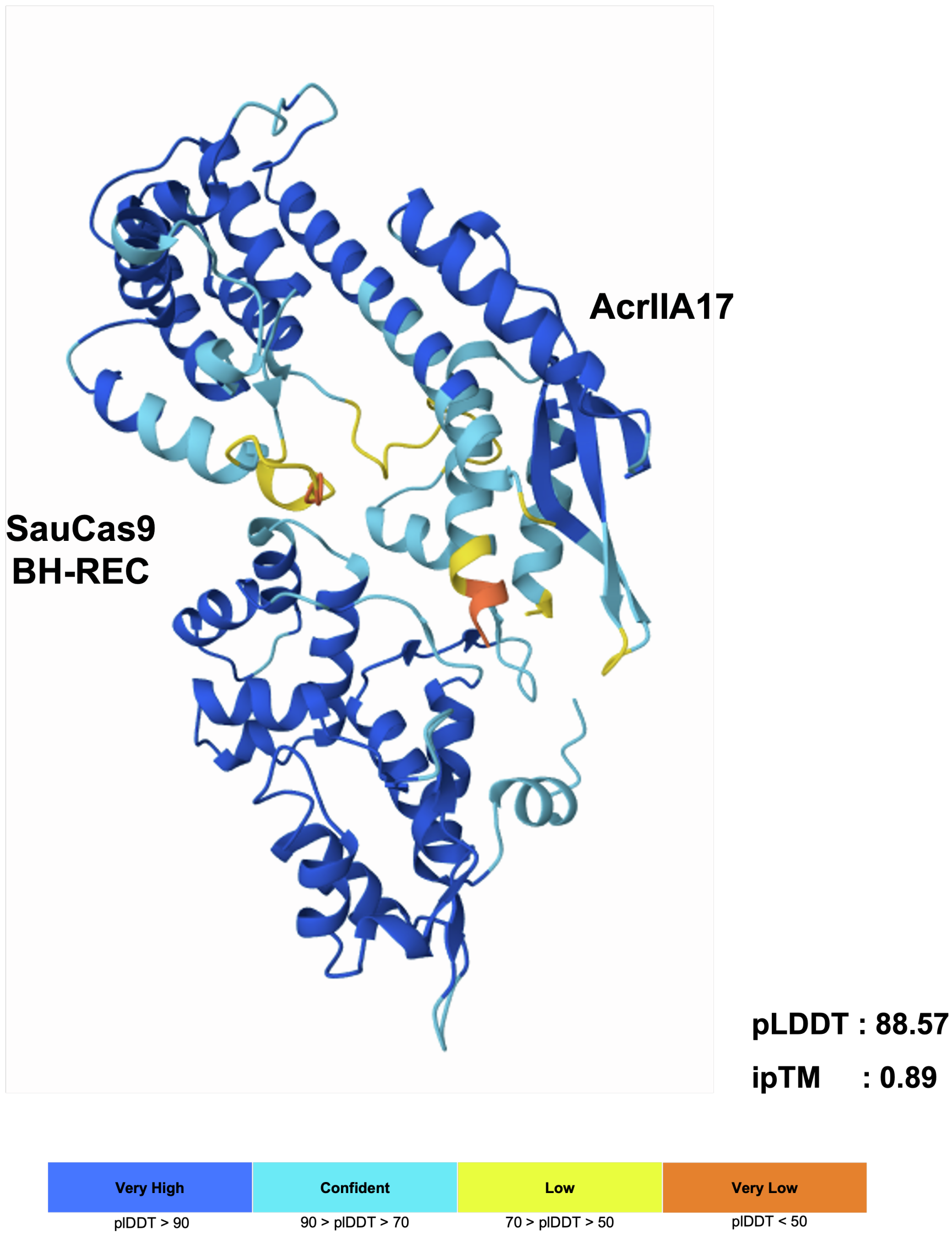

Supplementary Fig. S1. AlphaFold3 model of the AcrIIA17–SauCas9 complex. The model is colored according to pLDDT confidence scores, ranging from very high confidence (blue) to very low confidence (orange). The overall model exhibited a mean pLDDT value of 88.57 and an ipTM score of 0.89, supporting a high-confidence prediction of the interaction interface.

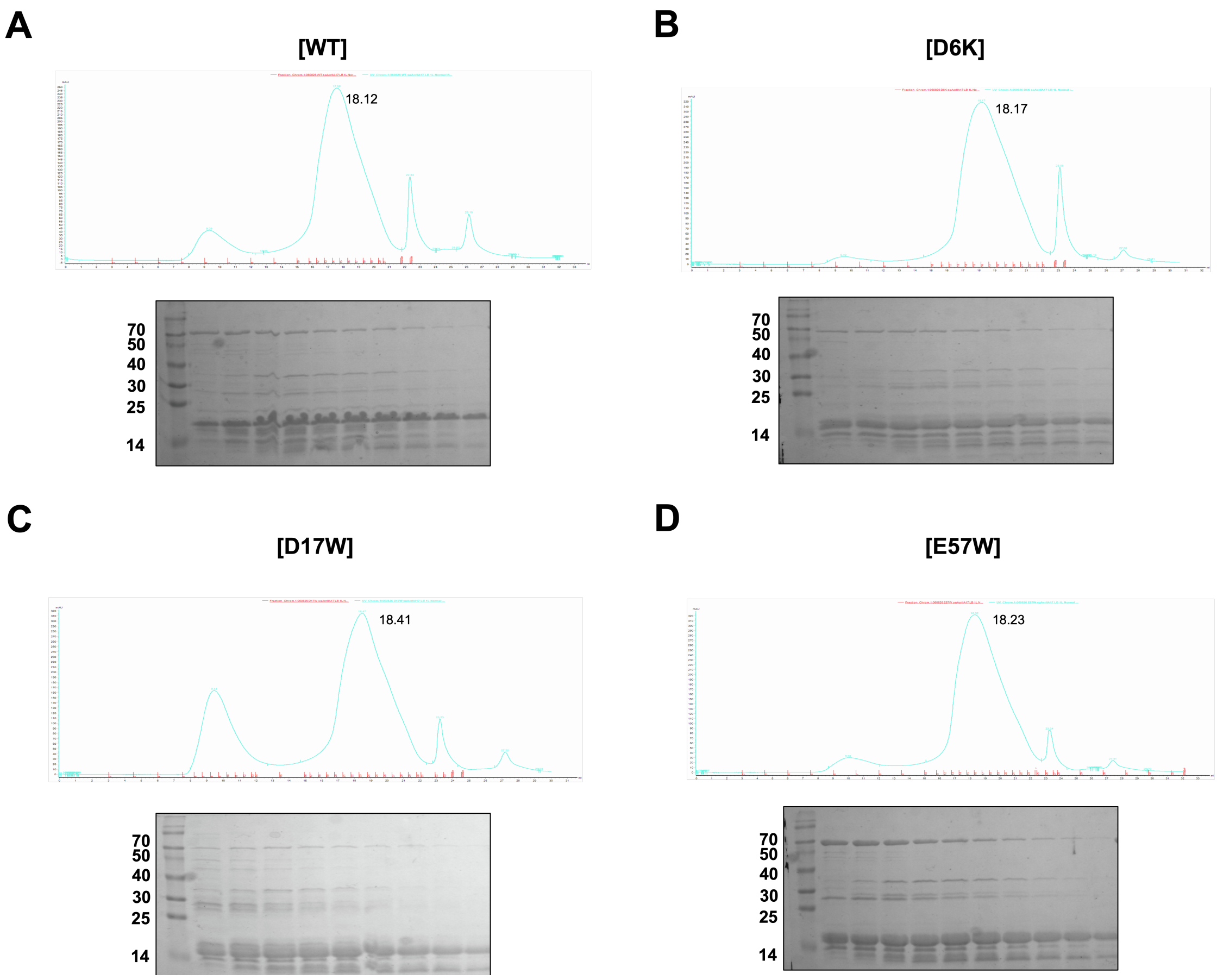

Supplementary Fig. S2. Purification and SEC analysis of wild-type and mutant AcrIIA17 proteins. (A) Wild-type AcrIIA17. (B) D6K mutant. (C) D17W mutant. (D) E57W mutant. Upper panels show size-exclusion chromatography (SEC) profiles, and lower panels show SDS-PAGE analysis of the corresponding purified proteins.

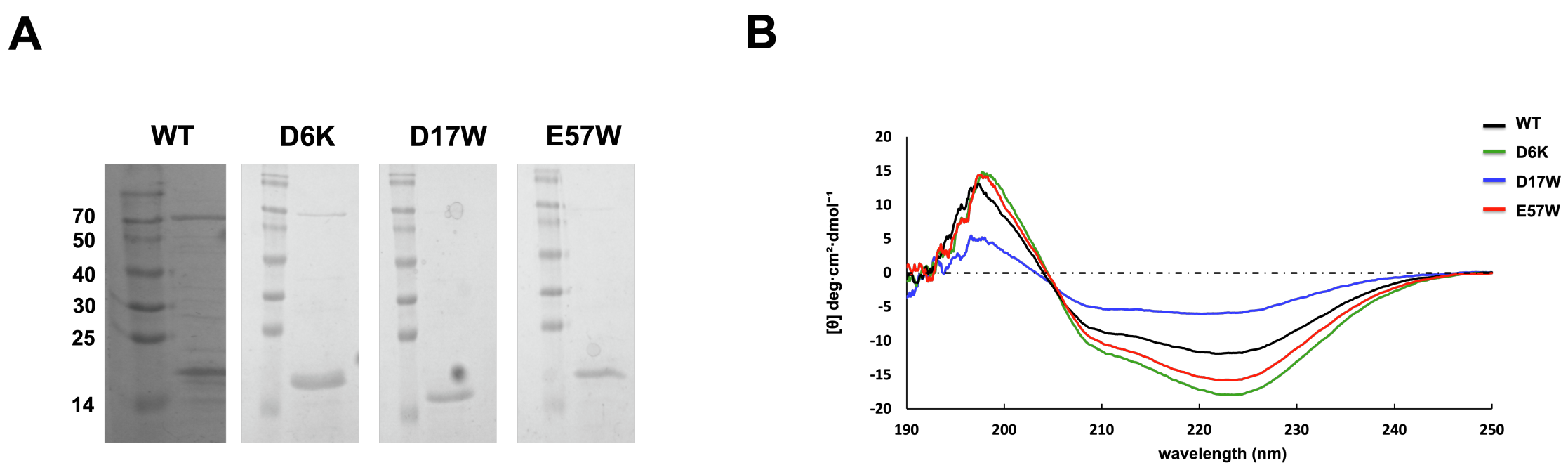

Supplementary Figure S3. Structural characterization of wild-type and mutant AcrIIA17 proteins. (A) SDS-PAGE analysis of purified wild-type, D6K, D17W, and E57W AcrIIA17 proteins. All proteins were purified to comparable homogeneity. (B) Circular dichroism (CD) spectra of wild-type and mutant AcrIIA17 proteins. The mutant proteins displayed CD spectra similar to that of wild-type AcrIIA17, indicating that the mutations did not cause significant alterations in secondary structure or global protein folding.

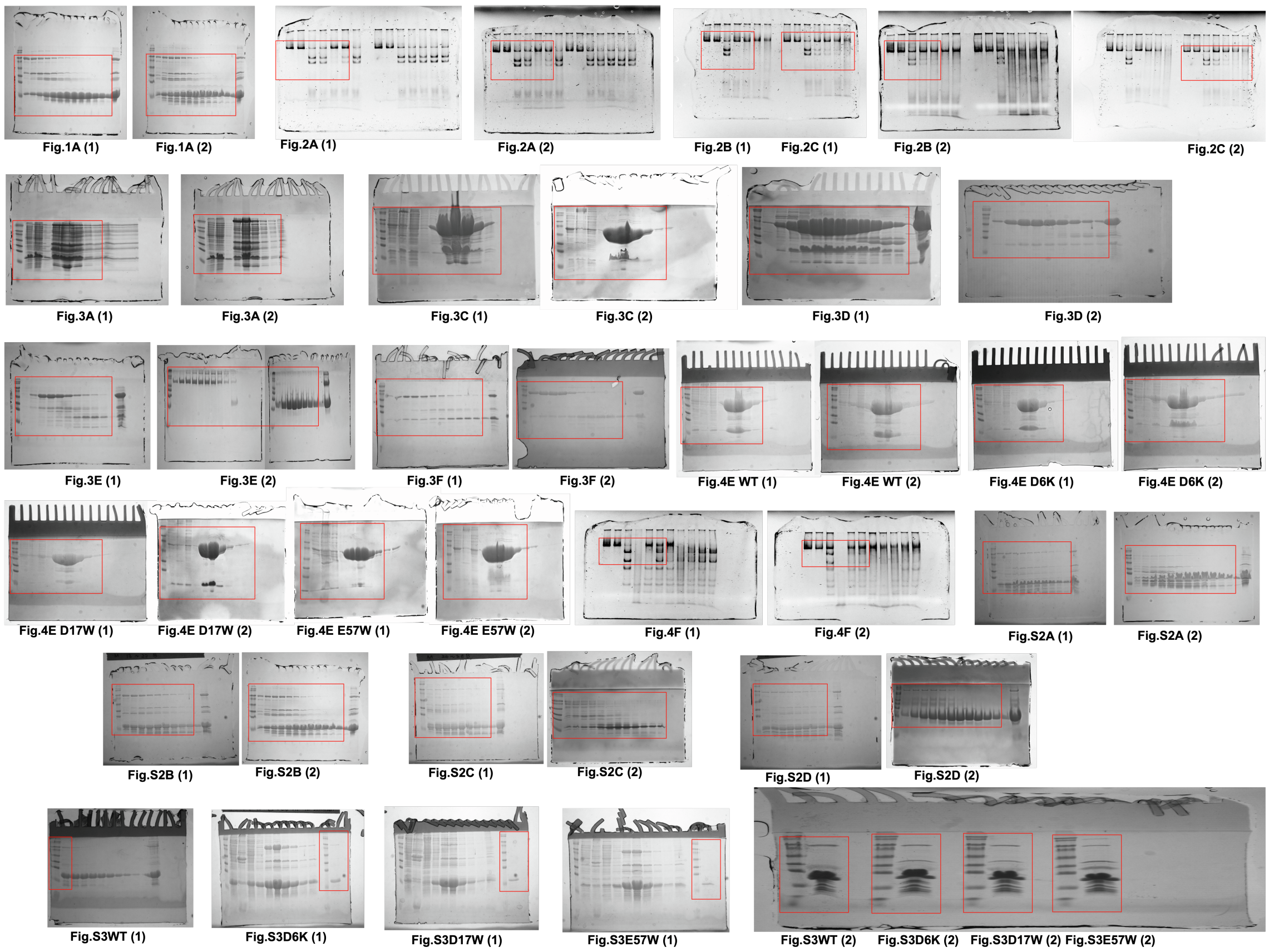

Supplementary Figure S4.Uncropped blot gel images. The red boxes indicate the portion that was cropped and used in the manuscript. All experiments were performed twice. The numbers in parentheses indicate the following: (1) the blot image presented in the main text as a representative experimental result, and (2) the blot image obtained from an independent repeat experiment performed to confirm the result.
